# Information Flow Network of International Exchange Rates and Influence of Currencies

**DOI:** 10.3390/e23121696

**Published:** 2021-12-18

**Authors:** Hongduo Cao, Fan Lin, Ying Li, Yiming Wu

**Affiliations:** Sun Yat-sen Business School, Sun Yat-sen University, Guangzhou 510275, China; linfan647@foxmail.com (F.L.); wuym28@mail2.sysu.edu.cn (Y.W.)

**Keywords:** exchange rate, currency relationships, complex network, transfer entropy, causality analysis

## Abstract

The main purpose of the study is to investigate how price fluctuations of a sovereign currency are transmitted among currencies and what network traits and currency relationships are formed in this process under the background of economic globalization. As a universal equivalent, currency with naturally owned network attributes has not been paid enough attention by the traditional exchange rate determination theories because of their overemphasis of the attribute of value measurement. Considering the network attribute of currency, the characteristics of the information flow network of exchange rate are extracted and analyzed in order to research the impact they have on each other among currencies. The information flow correlation network between currencies is researched from 2007 to 2019 based on data from 30 currencies. A transfer entropy is used to measure the nonlinear information flow between currencies, and complex network indexes such as average shortest path and aggregation coefficient are used to analyze the macroscopic topology characteristics and key nodes of information flow-associated network. It was found that there may be strong information exchange between currencies when the overall market price fluctuates violently. Commodity currencies and currencies of major countries have great influence in the network, and local fluctuations may result in increased risks in the overall exchange rate market. Therefore, it is necessary to monitor exchange rate fluctuations of relevant currencies in order to prevent risks in advance. The network characteristics and evolution of major currencies are revealed, and the influence of a currency in the international money market can be evaluated based on the characteristics of the network. The world monetary system is developing towards diversification, and the currency of developing countries is becoming more and more important. Taking CNY as an example, it was found that the international influence of CNY has increased, although without advantage over other major international currencies since 2015, and this trend continues even if there are trade frictions between China and the United States.

## 1. Introduction

Exchange rate determination theories have gradually formed theories such as the purchasing power parity theory reflecting commodity market equilibrium; the interest rate parity theory reflecting the asset market equilibrium; and the dynamic stochastic general equilibrium model (DSGE) of exchange rates, which integrates these two preconditions into a general equilibrium analysis of exchange rate determination [1,2,3,4]. Based on the bilateral relationship analysis of currency, the traditional exchange rate determination theory emphasizes the value attribute of currency as a general equivalent but ignores the natural network attribute of currency.

In a free trade and open capital market environment, the overall geometric characteristics of the exchange rate network reflect market equilibrium, while the individual network characteristics of each currency reflect the status of the currency in the international monetary system and its influence on other currencies. The final formation of the equilibrium exchange rate of each sovereign currency is not only the result of each country’s exchange rate policy, macroeconomics and trade structure but also inevitably affected by the overall characteristics of the exchange rate correlation network and the individual characteristics of each country’s currency in the network. The analysis of the network characteristics of currency is undoubtedly important and valuable.

Under the background of trade and financial globalization, international trade and capital flows are becoming increasingly frequent, and the fluctuation of foreign exchange market is increasing. The exchange rate market has very obvious network characteristics, and multilateral exchange rates affect each other, making the butterfly effect possible. For example, Brexit resulted in a chain reaction of major currencies such as GBP, EUR, USD, JPY and CNY and further caused turmoil in the international monetary market. As foreign exchange market risks continue to increase, there will be knock-on effects and risk contagion in most economies around the world. The spatial interaction of the exchange rate returns of various currencies has increased and presents multi-dimensional, mixed and asymmetric characteristics [5,6,7,8,9]. These new characteristics improve the instability of the global exchange rate market. 

There are not only direct interactions among multiple variables but also indirect interactions with intermediate variables as bridges, and the influence relationship is usually asymmetric. The traditional correlation analysis method has difficulty confronting indirect relationships and asymmetric influence relationships, which are very limited in practical applications. With an increase in system complexity, causality analysis method has attracted extensive attention. In 1969, Granger first proposed the Granger causality analysis method, which has been widely used in many fields [10]. The Granger causality analysis method can only provide qualitative analysis results in spite of having strong interpretability, and it is easy to produce false causality for high-dimensional time series due to its basing on linear models. Information-theoretic approaches are important means for analyzing information flow between two or more systems. After the concept of transfer entropy was put forward, various types of causality analysis models based on information theory appeared [11]. Based on the basic framework of information theory, transfer entropy is a nonparametric model method, which can well analyze the coupling strength and asymmetric driving response relationship of the two systems. Barnett et al. proved that Granger causality analysis and transfer entropy are equivalent when variables obey Gaussian distribution [12]. Moreover, the measurement of information flow is directional and can identify the direction of fluctuation transmission. 

The fact of neglecting the network characteristics of currency implies that the traditional monetary theory holds that there is no obvious difference between the network characteristics of different currencies, and the roles of different currencies are also similar in the global transmission of exchange rate fluctuation. It is necessary to investigate how the price fluctuation of a sovereign currency is transmitted among currencies and what network traits and currency relationships are formed in this process under the background of economic globalization. This paper introduces information theory and transfer entropy to measure information transmission between exchange rate fluctuations and the interaction between currencies. The directed weighted information flow network is constructed to reveal and analyze the interaction between global currencies and the evolution characteristics of the influence of major currencies, particularly the static characteristics and dynamic evolution of CNY international influence.

This paper is organized as follows. The second section provides a literature review of relevant theories and applications. The third section examines exchange rate network construction and analysis method based on transfer entropy. The fourth section provides empirical analysis of an exchange rate network. The fifth section is the conclusion.

## 2. Literature Review

A complex network is a useful tool for studying complex systems with a large number of individuals. The question of how to measure correlations between nodes is the first issue considered in network construction. Pearson correlation coefficient, as the simplest method to measure the correlation of nodes, is widely used by scholars around the world. However, this method is based on Gaussian distribution and linear correlation, so it is not effective in processing data with thick tails, volatility clustering, frequent extremes and other characteristics. Dias and Embrechts (2010) used the time-varying Copula-GARCH model to measure the correlation between the exchange rate of EUR against USD and the exchange rate of JPY against USD and found that the tail dependence between currencies using the non-time-varying model would be overestimated [13]. A time-varying model is complicated, difficult to apply in a wide range and can only deal with a small amount of exchange rate time series. Tiange Wen etc. (2020) used the extended model of LASSO-VAR to construct a high-dimensional volatility spillover network of 52 kinds of encrypted currencies; LASSO-VAR is more effective in processing high-frequency data [14]. In addition, Granger causality test and other methods are often used to measure time series correlations [15].

Based on information theory, mutual information can reflect nonlinear correlations between two time series and measure information flow between the series. Fiedor (2014) used Lempel–Ziv complexity to estimate mutual information and mutual information rates and further used it as correlation measure to study the financial market. Two methods, MST and PMFG, were used to build the network, which was compared with the network established by Pearson correlation coefficient from three aspects: node, clustering coefficient and network structure. It was proved that the network established by mutual information is better [16]. Tao and Hołda (2015) used information entropy to estimate partial mutual information and measure the correlation between nodes and found that there was no structural risk in China ‘s stock market [17]. Hu etc. (2018) based on the study of public financial institutions in China established a mutual information coefficient network model, and it was proved that correlation enhancement can improve the systemic risk of financial institutions. However, it is not completely synchronized, and correlation can be used to predict the emergence of systemic risks [18].

The calculation methods of mutual information mainly include Lempel–Ziv complexity and information entropy. Transfer entropy is a derivative index in the concept of information entropy. Due to directionality, transfer entropy plays a significant role in exploring the contagion mechanisms of financial crisis and volatility transmission. Wu Songtao and He Jianmin (2018) measured information flow between stock markets by transfer entropy, which helps to identify nonlinear relationships [19]. Oh, etc., (2014) and Bekiros, etc., (2017) used transfer entropy to measure changes in stock market information flow before and after the financial crisis [20,21]. Junior, etc., (2015) and Kwon and Oh (2012) used transfer entropy and found that the information flow between the stock indexes of different countries is asymmetrical. The above research studies show that transfer entropy can well measure the different temporal and spatial characteristics of stock market information flow before and after key events in terms of intensity and direction [22,23]. Yang, etc., (2014) used the mutual information method and set a sliding window to further investigate the risk contagion among American industrial sectors by using the method of transfer entropy. The research results found that the financial sector was the source of risk transmission and seriously affected energy, raw materials and industrial industries [24]. Thomas, etc., (2013) measured information flow among financial markets by using transfer entropy, tested the importance of credit risk pricing in the credit default swap market relative to the corporate bond market and analyzed the dynamic relationship between market risk and credit risk represented by the VIX and European iTraxx index [25].

## 3. Construction and Analysis of Exchange Rate Network Based on Transfer Entropy

This section may be divided by subheadings. It should provide a concise and precise description of the experimental results, their interpretation, as well as the experimental conclusions that can be drawn.

### 3.1. Basic Theory

Shannon (1948) proposed the concept of information entropy, which laid the foundation for the establishment of information theory [26]. The concept of information flow was first derived from computer systems and communication networks, and then it was used in other fields. Since information flow can better capture nonlinear correlations between systems by using the concept of entropy, it has unique advantages in studying the correlation of time series. In today’s society, trade, capital flow and financial lending have resulted in information flow between financial markets, and information flow has the characteristics of being aggregated and traceable; thus, it is widely used in the field of exploring the contagion mechanism of financial crises.

Entropy *H*(*X*) is used to measure the amount of information of a single discrete random variable *X*. The calculation formula is as follows.

(1) $$H\left(X\right)=-\underset{x}{\sum }p\left(x\right)log_{2}p\left(x\right)$$

(2) $$H\left(X\right)=-\sum_{x}p\left(x\right)log_{2}p\left(x\right)\;\;H\left(X,Y\right)=-\sum_{x}p\left(x,y\right)log_{2}p\left(x,y\right)$$

*p*(*x*,*y*) is the joint distribution of discrete random variables $\left(X,Y\right)$. Conditional entropy *H*(*X*|*Y*) can measure the uncertainty of *X* when *Y* is determined.

(3) $$H\left(X|Y\right)=-\underset{x}{\sum }p\left(x,y\right)log_{2}p(x|y)$$

Based on the above concept, Schreiber (2000) proposed the concept of transfer entropy and used it to measure asymmetric information flow between systems [27]. For the two systems, *I* and *J* are expressed as $I:\left\{i_{n},n=1,2,3\ldots ,N\right\}$ and $J:\left\{j_{n},n=1,2,3\ldots ,N\right\}$; $i_{n}$ and $j_{n}$ are the states of system *I* and *J* at time n; the information transfer quantity *TE* of system *J* on system *I* can be expressed by transfer entropy; and the calculation formula is described as follows:

(4) $$TE_{J\to I}\left(k,l\right)=H_{I}\left(k\right)-H_{IJ}\left(k,l\right)=\sum p\left(i_{n+1},i_{n}^{k},j_{n}^{l}\right)log_{2}\frac{p(i_{n+1}|i_{n}^{k},j_{n}^{l})}{p(i_{n+1}|i_{n}^{k})}$$

where $H_{I}\left(k\right)=-\sum p\left(i_{n+1},i_{n}^{k},j_{n}^{l}\right)log_{2}p(i_{n+1}|i_{n}^{k})$ is the conditional entropy of k-order delay subsequence $i_{n}^{k}=\left(i_{n},\ldots ,i_{n-k+1}\right)$ of the known system. $H_{IJ}\left(k,l\right)=-\sum p\left(i_{n+1},i_{n}^{k},j_{n}^{l}\right)log_{2}p(i_{n+1}|i_{n}^{k},j_{n}^{l})$ is the conditional entropy of k-order delay subsequence of the known system *I* and l-order delay subsequence $j_{n}^{l}=\left(j_{n},\ldots ,j_{n-l+1}\right)$ of the known system *J*; $p\left(*\right)$ is the probability of state occurrence. We set k=l=1 [28], then the probability distribution of the random variable can be obtained for calculating transfer entropy. Transfer entropy is directional: $TE_{J\to I}\neq TE_{I\to J}$. If $TE_{J\to I}>TE_{I\to J}$. It indicates that the amount of information transmitted by J system to I system is relatively large, and J system is the information source of I system.

### 3.2. Computing Processes

Before calculating transfer entropy, we first calculate the logarithmic return sequence of the daily frequency exchange rate of currency *i*, which is $R_{i}\left(t\right)=\ln P_{i}\left(t+1\right)-\ln P_{i}\left(t\right)$. Secondly, the symbolic coding method is used [29]. According to the third quantiles, the sequence of returns is divided into three states, and the set of states is $s=\left\{1,2,3\right\}$, the logarithmic return sequence of the daily frequency exchange rate of currency *i* can be transformed into a discrete state sequence according to Formula (5), and the frequency of each state can be approximated as a probability distribution to calculate transfer entropy TE.

(5) $$s_{x}=\left\{\begin{array}{l} 1\;\;\;\;\;\;\;\;\;\;\;\;\;\;\;\;\;\;\;R_{i}\left(t\right)<q_{\frac{1}{3}}\\ 2\;\;\;\;\;\;\;\;\;q_{\frac{1}{3}}<R_{i}\left(t\right)<q_{\frac{2}{3}}\\ 3\;\;\;\;\;\;\;\;\;\;\;\;\;\;\;\;\;\;\;q_{\frac{2}{3}}<R_{i}\left(t\right) \end{array}\right.$$

Based on the above discrete state sequence, the transfer entropy between currencies under each time window length was calculated with *R* software according to formula (4), and the total output value and total input value of each currency’s transfer entropy are calculated by Formulas (6) and (7).

(6) $$TE_{total-out}=\overset{29}{\underset{i=1}{\sum }}TE_{J\to I_{i}}$$

(7) $$TE_{total-in}=\overset{29}{\underset{i=1}{\sum }}TE_{I_{i}\to J}$$

The calculation formula for the elements in the transfer entropy matrix is described in Formula (8).

(8) $$c_{ij}=TE_{I\to J}$$

Static analysis uses the threshold method to build a network, and the specific process is described as follows: Firstly, calculate an optimal threshold [30,31]. The calculation formula of this threshold is $W\left(N,R^{2}\right)=\left(N/N_{all}\right)\times R^{2}$. $N/N_{all}$ represents the number of nodes in the network divided by the total number of nodes. When the threshold increases, individual nodes in the network become isolated points that destroy the network’s structure. $N/N_{all}$ is used to measure the integrity of the network. Barabasi (1999) pointed out that the power law is a sign of self-organization of complex systems, which can measure the geometric invariance of the power rate to a certain extent. If the threshold increases, it helps to improve the fitting degree of the power law of the network [32]. The optimal threshold is set to achieve a balance between preserving network integrity and improving the fitting degree of power law in order to determine threshold θ. After the threshold is selected, if the element $c_{ij}\geq \theta$ in the matrix C is formed by the transfer entropy, then node i and node j are connected in the network, and $A_{ij}$ is assigned to 1 in the adjacency matrix $A\left(N\times N\right)$. If $c_{ij}<\theta$, there is no connection between node i and node j in the network, and $A_{ij}$ is assigned to 0.

(9) $${\left(A_{all}\right)}_{ij}=\left\{\begin{array}{l} 1,\;\;\;|{\left(C_{all}\right)}_{ij}|\geq \theta \\ 0,\;\;\;|{\left(C_{all}\right)}_{ij}|<\theta \end{array}\right.$$

### 3.3. Exchange Rate Network Characteristic Index

#### 3.3.1. Centrality Index

Centrality index can be divided into degree centrality, betweenness centrality and closeness centrality. Suppose $\omega_{ij}$ is the weight of the connection from node *i* to node *j*, and the value is the product of the amount of information and the elements at the corresponding position of the adjacency matrix, such as $\omega_{\mathrm{ij}}=c_{\mathrm{ij}}\times A_{\mathrm{ij}}$.

The weighted absolute degree centrality of node *i* $De_{i}$ *i* is described by Formula (10).

(10) $$De_{i}=\overset{N}{\underset{j=1}{\sum }}\omega_{ij}{}_{i}$$

In the directed network, degree centrality is further divided into weighted output degree and weighted input degree according to the different directions. The weighted output degree calculates the sum of the output value from node *i* to other nodes: $\sum_{j=1}^{N}\omega_{i\to j}{}_{i}\left(j\neq i\right)$. The weighted input degree calculates the sum of the input values from other nodes to node *i*: $\sum_{j=1}^{N}\omega_{j\to i}{}_{i}\left(j\neq i\right)$. Net flow degree is defined as the difference between the weighted output and input degree: $\sum_{j=1}^{N}\omega_{i\to j}{}_{i}-\sum_{j=1}^{N}\omega_{j\to i}{}_{i}\left(j\neq i\right)$.

Betweenness centrality is used to measure the connectivity of nodes in the network. It is assumed that the number of shortcuts between node *j* and node *k* is $Dis_{jk}$, in which the number of passing through point *i* is $G_{jk}\left(i\right)$. The absolute betweenness centrality $Be_{i}\;$ equals the ratio of the number of shortcuts passing through node *i* to the total number of shortcuts between node *j* and node *k*.

(11) $$Be_{i}=\underset{j,k}{\sum }G_{jk}\left(i\right)/Dis_{jk}$$

Closeness centrality is used to measure the control power of nodes in the network. Some nodes have few connections in the network, but the distance with other nodes is shorter; these nodes can still affect closer nodes. The sum of the shortest distance $d_{ij}$ between node *i* and all other nodes in the figure is the absolute closeness centrality. In the directed network, this indicator needs to distinguish directions, and relative closeness centrality $Ce_{i}$ is the reciprocal of normalized absolute closeness centrality.

(12) $$Ce_{i}=\frac{N-1}{\sum_{j=1}^{n}d_{ij}}\times 100\%$$

#### 3.3.2. PageRank Value

PageRank algorithm is an algorithm used by the Google search engine to measure the importance of web pages, and it is also an effective method for evaluating the importance of nodes in directed networks. The PageRank algorithm, in measuring the importance of nodes, takes both the output degree and input degree of nodes into account. The calculation formula of the index is described as follows.

(13) $$PR\left(A\right)=\left(1-d\right)+d\left(\frac{PR\left(T_{1}\right)}{C\left(T_{1}\right)}+\ldots +\frac{PR\left(T_{N}\right)}{C\left(T_{N}\right)}\right)$$

$PR\left(A\right)$ is the PageRank value of node A; $R\left(T_{i}\right)$ is the PageRank value linked to node A; $C\left(T_{i}\right)$ is the output degree of node *i*; and *d* is damping coefficient as an adjustable parameter, 0<d<1. Formula (13) shows that the PageRank value of node *A* is largely related to the PageRank value of the other nodes pointing to it; that is, the importance of nodes in the network depends on the number of links by other nodes.

### 3.4. Blockmodels Analysis

White etc. (1976) proposed a block-modeling method for analyzing different blocks and roles in complex networks [33]. The CONCOR method is the most commonly used method. The calculation steps are as follows. Firstly, the correlation coefficient between rows and columns of the input matrix C is calculated in order to obtain correlation coefficient matrix C1. Then, C1 is used as an input matrix to calculate correlation coefficient matrix C2 between rows and columns. After repeated calculations, a correlation coefficient matrix $C_{\mathrm{end}}$ consisting of only 1 and −1 is generated [34]. After rearranging the elements of the $C_{\mathrm{end}}$ matrix, the matrix can be partitioned. CONCOR uses a tree diagram to display the specific conditions of the blocks and mark the network members owned by each block. Wasserman and Faust (1994) [35] proposed evaluation indicators of internal and external network relationships to judge the role of blocks, as shown in Table 1. Assuming that there are $g_{k}$ subjects in block $A_{k}$, the total number of relationships theoretically existing in the block is $g_{k}\left(g_{k}-1\right)$. If there are *g* agents in the entire network, the total number of theoretical relationships between agents in the block $A_{k}$ is $g_{k}\left(g-1\right)$. Nodes in the network can be roughly divided into four blocks: The first is the primary block in which the members send more information to the inside and less information to the outside but also receives information from other blocks. The second is the sycophants, in which the members send less information to the internals and more information to the externals and have spillover effects on other blocks but receives less information from external blocks. The third includes the isolates, and their members send more information both internally and externally but receive less information from the outside. The fourth is the brokers, in which the members receive and send information from other blocks at the same time, less information is received from internal members, and it mainly undertakes the intermediary role of information transmission in the block.

## 4. Empirical Results of Exchange Rate Network

### 4.1. Data Sources and Network Building Process

The data are from the IMF statistical database, which includes daily exchange rate data for 30 countries or regions from 1 January 2007 to 31 December 2019, with exchange rate benchmarks in SDRs. Currency abbreviations for 30 countries or regions, English names and regions are shown in Table 2. In this paper, static and dynamic analyses of exchange rate information flow network are carried out, respectively. The daily frequency exchange rate data of 30 countries or regions from 1 January 2007 to 31 December 2019 were used to calculate the overall transfer entropy matrix C_total by counting 3276 sample points. The eigenvalue changes of the annual information network were calculated. The sample length of the annual exchange rate information network was 252 days, and the annual transfer entropy matrix C_ti (ti = 1, 2 … 13) was calculated.

In this paper, the ADF test was used to test the stationarity of the logarithmic first-order difference of the daily frequency exchange rate series. The *p*-values are all less than 1%; thus, the data are stable and can be used for subsequent analysis. After transforming the rate of return on the currency exchange rate into a discrete time series, the total output value and total input transfer entropy value of each currency are calculated by using Formulas (6) and (7).

### 4.2. Static Analysis of the Eigenvalues of the Overall Exchange Rate Network

**Figure 1:**
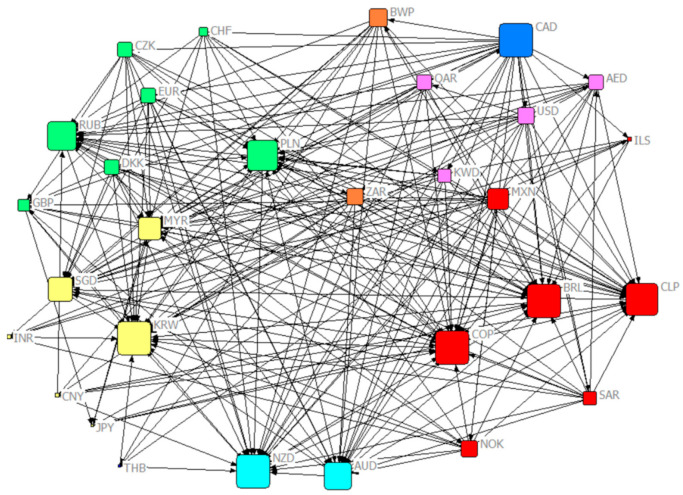
Weighted directed overall exchange rate threshold network (the threshold is 0.23).

Based on the overall transfer entropy matrix $C_{total}$, a threshold network was established, as shown in Figure 1. The size of the node represents the node degree (the sum of output and input degree). The currencies with higher degrees are the Canadian dollar, Korean dollar, Australian dollar and Russian rupee. This paper further calculates the weighted centrality index and Pagerank ranking of nodes, and the results are shown in Table 3.

#### 4.2.1. Weighted Input Degree

A node with a higher weighted input degree indicates that it receives a larger amount of information. There are two main categories: The first category is the currencies with commodity attributes, including South American currency, Brazilian real, Chilean peso, Colombian peso, Russian ruble, Australian dollar, New Zealand dollar and so on. Commodity currencies refer to legal currencies with certain commodity attributes. Most of the countries in which such currencies belong have strategic resources such as oil, coal and minerals and rely on the export of raw materials to develop the economy. The currency exchange rate is generally highly correlated with the commodity price of a certain export that accounts for a higher proportion of GDP. Common commodity currencies include Australian dollar, Canadian dollar, New Zealand dollar, South Africa and Norwegian krone. The other category is the currency of transportation hub countries, including Singapore dollar, Poland zloty and Malaysian ringgit. The countries where these currencies belong have a special geographical location and are important transit points in international trade. Commodity exports increase the economic dependence of the above countries on exporters. Therefore, when the exchange rate of exporters fluctuates, trade becomes the medium of volatility transmission, resulting in an increase in information flow received by the currency, which has an impact on the domestic exchange rate.

#### 4.2.2. Weighted Output Degree

The nodes with highly weighted output degrees indicate that they have a large amount of information transmitted outward, which mainly include African currencies (BWP and ZAR), American currencies (Canadian dollar and Mexican peso) and European currencies (from large to small, followed by Norwegian kroner, Poland zloty, Euro and Danish kroner). It can be observed that the proportion of commodity currency in the nodes with highly weighted output degrees is still high, and the information flow output by BWP in the network is also high because of the crawling peg to South African rand. In addition, EUR has strengthened the integration of European countries’ trade and finance, improved the international status of each member country and enhanced the influence of the European foreign exchange market. Therefore, the transfer entropy output value of European currencies is relatively high.

#### 4.2.3. Weighted Closeness Centrality

The higher weighted input closeness centrality indicates that it is easier for other nodes in the network to reach this node. Table 4 shows that the higher nodes almost had relatively higher weighted input degrees. The higher weighted output closeness centrality indicates that it is easier for this node in the network to reach other nodes. The currencies with relatively higher index are mainly the oil currency group represented by the US dollar (USD, Kuwaiti dinar, Saudi Arabian rial, AED and QAR) and the currencies with higher weighted output degree (CAD, Swiss franc, Poland zloty and Czech Republic). Although the weighted out degree of the Singapore dollar is low, it has a strong influence because of its special geographical location and international status in the network and its short distance from other nodes.

#### 4.2.4. Betweenness Centrality

The betweenness centrality values show obvious stratification, and the higher nodes are mainly the Canadian dollar and Singapore dollar. The importance of the Singapore dollar has already been analyzed in the previous article. It has a high betweenness centrality of the country as a trading hub. Canada’s economy is strongly dependent on trade exports and mainly exports agricultural products, marine products and oil. Commodity exports are an important source of income for Canada. Rising or falling commodity prices usually result in Canadian dollar appreciation or depreciation and trigger a chain reaction in the global commodity market. Therefore, as one of the most active commodity currencies in the world, the Canadian dollar has high values for the above four indicators and is at the core of the network.

#### 4.2.5. PageRank Value

Currencies with higher PageRank value indicate that they play an important role in a directed network, which can be divided into two categories: the first category is currencies with commodity attributes represented by Brazilian real, Chilean peso, Colombian peso, Russian ruble and Canadian dollar. This phenomenon shows that currency is an important medium for the flow of commodities, and the globalization of trade has enhanced the transmission of information between currencies and strengthened the linkage of the foreign exchange market. The second category is the currencies of developed countries such as the U.S. dollar and the euro. These countries or regions have relatively strong economic strength, more open trade and more developed capital markets, and they have a quicker process of internationalization. Therefore, they have become the information source in the network with strong anti-interference ability, and the information flow sent is much larger than the information flow received.

The connection of CNY in the network is lesser, and the output degree is six, connected to RUB, Colombia peso, Chile peso, BRL, Poland zloty and New Zealand dollar. Its input degree is one, connected by South Africa. As a major trading country, China’s demand for energy, minerals and primary raw materials can affect the global market. At the same time, CNY is also one of the world’s main settlement currencies; thus, it has a certain influence in the network. However, the PageRank value of CNY is low, and its influence is limited mainly for currencies of emerging economies.

### 4.3. Blockmodels Analysis

**Figure 2:**
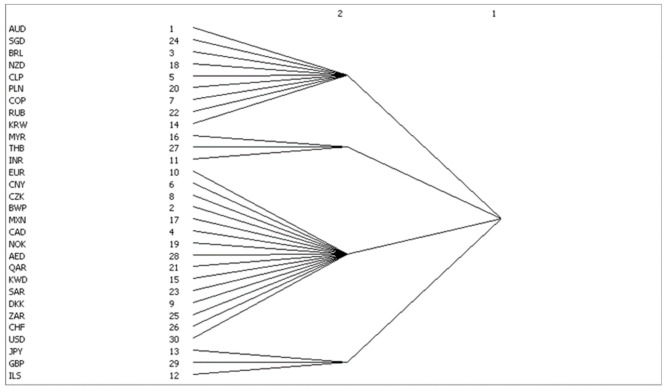
The blocks divided by CONCOR.

In this paper, UCINET is used to analyze the network block model, and the results are shown in Figure 2. The first block consists mainly of commodity currencies (AUD, New Zealand dollar and RUB), transport hub currencies (Singapore dollar and Poland zloty) and some South American currencies with commodity attributes (BRL, Chilean peso and Colombia peso). The second sector is composed of Malaysian ringgit, Thai baht and Indian rupee. The third sector consists of Euro zone currencies (Euro, Norway, Czech Republic, Denmark and Swiss franc), oil currency groups pegged to the US dollar (US dollars, Kuwaiti dinars, Saudi Arabia riyals, United Arab Emirates Diram and Qatar riyals), CNY, Canadian dollars, Mexican peso, BWP and South Africa. The fourth sector has three independent currencies: the yen, the pound sterling and the Israeli new shekel.

Table 4 shows that there are 201 correlations among the four blocks, in which 37 are within the block and 162 are between the blocks. It can be observed that there is a strong spillover effect between the blocks. The number of relationships issued by the first block is 26, of which 23 are within the block and 139 are received from the external block. According to the Table 1, this block belongs to the primary block. There are four associations issued by the second block to the outside, and the number of relationships within it is zero. There are 18 relationships with others, and they belong to the brokers. The third sent 159 relations, the number of internal relations is 14 and the received external relation is one, belonging to the sycophants. The fourth has 12 relationships, 0 internal relationships and 6 relationships with external blocks. It also belongs to the brokers.

The information source of the exchange rate market is mainly from the third block, which directly affects the first block and spills out excess information through the second block. The members of the fourth block are independent and do not interfere with each other, but they act as a bridge in the transmission of information. The Japanese yen in the fourth block is a typical arbitrage currency that can serve as a link to transfer external information to the currencies in the third block. Therefore, it can be inferred that the fluctuation of commodity currency is not only affected by the change of international trade and market demand but also related to arbitrage behavior.

### 4.4. Dynamic Analysis of Eigenvalue of Exchange Rate Network

The daily frequency exchange rate data of 30 countries or regions from 1 January 2007 to 31 December 2019 were used to calculate the eigenvalue change of annual information network. The sample length of the annual exchange rate information network was 252 days, and the annual transfer entropy matrix C_ti (ti = 1, 2 …13) was calculated. Based on the transfer entropy matrix with annual frequency, a dynamic weighted network was established, and the eigenvalues of the weighted network were analyzed. The dynamic statistical characteristics of the input (output) transfer entropy of each currency are shown in Table 5.

#### 4.4.1. Dynamic Analysis of Weighted Input Degree

**Figure 3:**
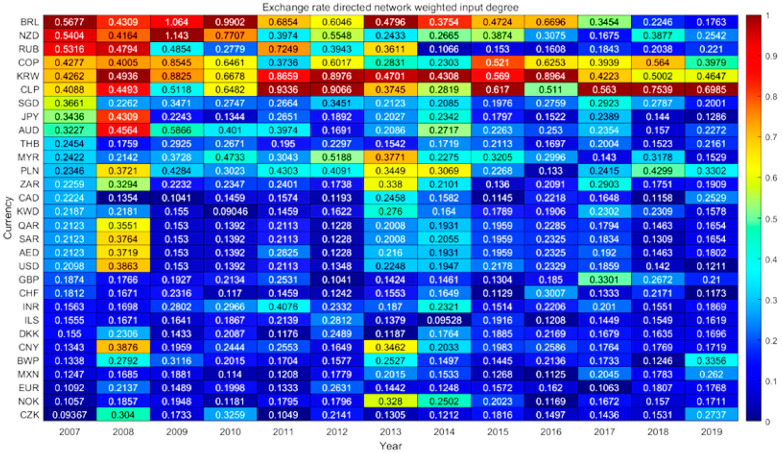
Exchange rate network weighted input degree heat map.

The dynamic results of weighted input degree of exchange rate information flow network are shown in Figure 3. The nodes with higher weighted input degree are currencies with commodity attributes and currencies of transportation hub countries. Horizontally, this paper finds that RUB’s weighted input degree has fallen sharply since 2014 and gradually rebounded until 2017. In 2014, the people of Crimea in Ukraine decided to leave Ukraine and enter Russia in the form of a referendum. The western countries led by the United States did not recognize it and, therefore, imposed sanctions on Russia. Economic and trade sanctions have had a huge impact on Russian ‘s international trade, capital flows and other aspects, and this has raised concerns about RUB’s credit standing, which may be the reason for blocking the flow of information between currencies resulting in a sharp decline in RUB’s weighted input degree. From the vertical point of view, the weighted input degree of US dollar-led oil currency increased during 2008. The financial crisis broke out in the United States and swept the world. All kinds of assets depreciated, capital was evacuated from the market and the increase in the demand for US dollar as a safe haven currency caused currency appreciation. In 2016, the Swiss franc also had a similar trend of change. In 2017–2019, CNY’s weighted input degree showed a downward trend.

#### 4.4.2. Dynamic Analysis of Weighted Output Degree

**Figure 4:**
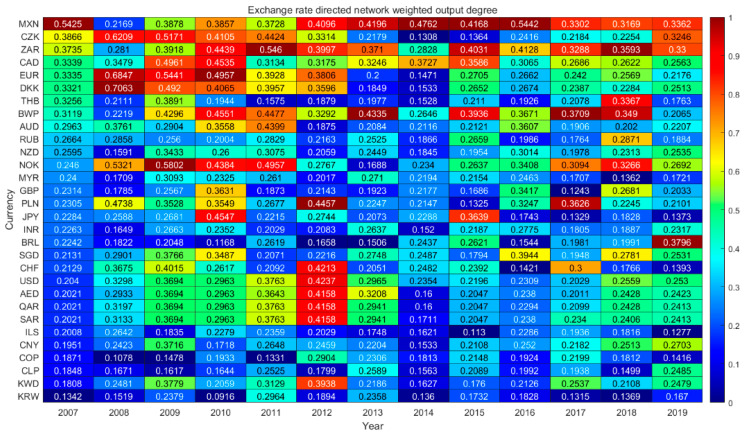
Exchange rate network weighted output degree heat map.

The dynamic analysis of weighted output degree of exchange rate information flow network is shown in Figure 4. The nodes with higher weighted output degrees are still the currencies with commodity attributes, such as the Norwegian kroner, the Canadian dollar, the South African rand and the Mexican peso. In addition, they also include European currencies (the Danish kroner and the Czech kroner). Horizontally, the weighted output degrees of the Euro, the Danish krone and the Czech krone have declined significantly since 2010. The escalating European debt crisis in 2010 and heightened market concerns resulted in a volatile decline in the euro. The crisis has not only caused sharp fluctuations in the Euro’s exchange rate, but it has also affected the Euro’s creditworthiness. According to 2012′s data, the holdings of Euro foreign exchange reserves held by central banks in developed countries increased by 15.8% over the previous year, but this increase was lower than the growth of used foreign exchange reserves, while developing countries continued to reduce their holdings of the euro. The debt crisis has damaged the Euro’s position in the global foreign exchange market, which is also reflected in the declining weighted output degree of the euro. CNY’s weighted output degree has declined significantly since 2017, mainly due to a weakening of its international influence as a result of the trade war. Vertically, the weighted output degree of safe-haven currencies such as the US dollar and Swiss franc increased in 2012. After the ups and downs of the European debt crisis in 2012, the third quarter ushered in a turning point, while the U.S. economy showed signs of recovery, and the Federal Reserve launched quantitative easing on several occasions to increase market confidence in the dollar. As central banks responded positively to the crisis, the US dollar, Swiss franc and others gained influence in the markets and are now sought after by safe-haven funds.

#### 4.4.3. Net Flow Degree

**Figure 5:**
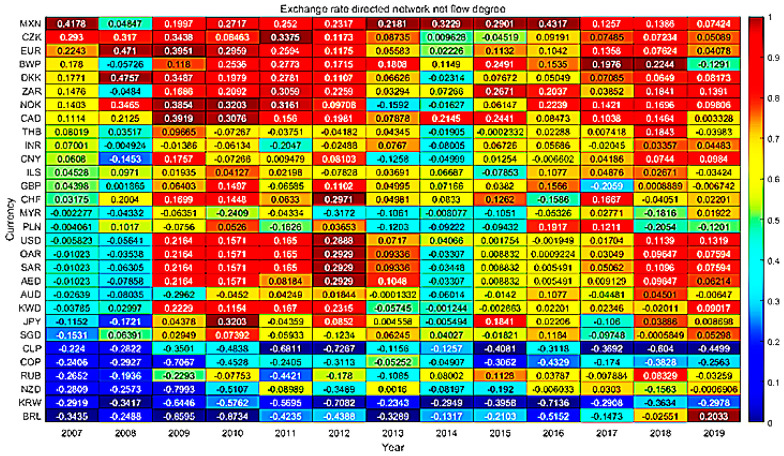
Exchange rate network net flow degree heat map.

Net flow degree is defined as the difference between the weighted output and input degree. Figure 5 shows that most of the nodes with negative net flow degree are currencies with commodity attributes, including South American currencies (Brazilian real, Chilean peso and Colombian peso) and Australian dollar, New Zealand dollar and Russian ruble. Commodity currency countries have long relied on export resources to drive GDP growth. Their currency trends and commodity trends are closely related to the rapid changes in the market; thus, volatility is large, and the amount of information received is greater than the amount of information transmitted. In the network, this plays the role of information sinks. Most of the larger net flow degrees include European currencies and oil currency groups represented by the United States dollar. The synergies in the EU have increased the influence of European currencies, and the US dollar, as an international reserve asset, tends to influence markets in its index movements; thus, these nodes exist as a source of information in the network. In addition, the net flow degrees of the ZAR, BWP, Norwegian krone and Canadian dollars are also high, indicating a larger flow of information sent by the above commodity currencies in the network. In addition, the Mexican peso, although not a commodity currency, has a high net flow degree, which is related to Mexico’s strong local influence in Latin America. In addition, the network of information sources in the period 2007–2012 was of high intensity and concentration, after which the information sources gradually began to disperse, and new sources such as the Thai baht, Indian rupee and CNY began to emerge in large numbers. The market is only affected by the single national currency that is gradually reduced, and a variety of forces emerging to compete for the foreign exchange market is the new normal.

### 4.5. Analysis of CNY Influence

**Figure 6:**
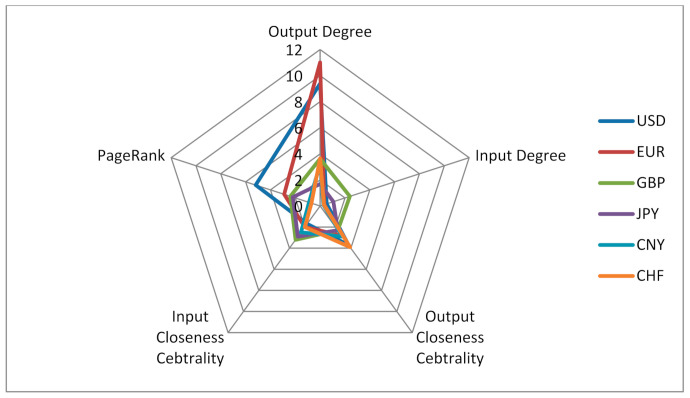
Radar chart of main currency influence from 2007 to 2019.

**Figure 7:**
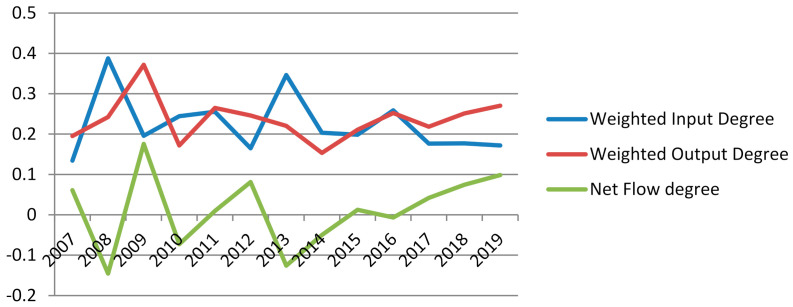
Dynamic characteristics of CNY influence index.

**Figure 8:**
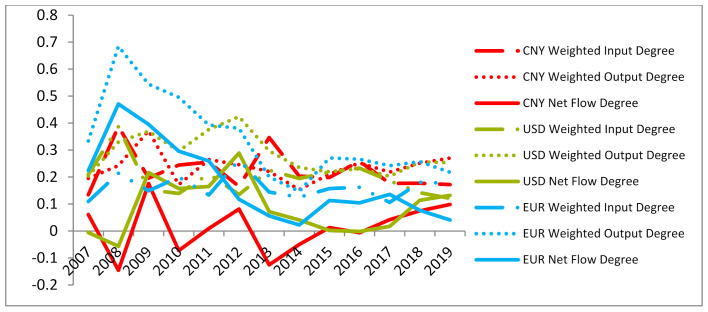
Dynamic comparison of CNY and US dollar and Euro influence index.

According to the weighted output degree, weighted input degree, weighted output closeness centrality, weighted input closeness centrality and PageRank of the monetary information correlation network from 2007 to 2019, the radar chart of CNY influence is established, as shown in Figure 6. It shows that, in the overall time span of 2007–2019, CNY has no advantages compared with major international currencies such as the United States dollar, the Euro, the pound, the yen and the Swiss franc. However, after the weighted output degree of CNY reached a minimum value in 2014, it gradually increased from 2015, and this trend was not affected by Sino–US trade frictions, as shown in Figure 7. The results show that the CNY “811” intermediate price quotation reform in 2015 and subsequent measures have contributed to the impact of CNY in the international monetary market. From the comprehensive influence reflected by the net flow degree, CNY surpassed the Euro for the first time in 2018, as shown in Figure 8.

### 4.6. Robust Analysis

In order to eliminate the influence of extreme minimum value on network construction, the 10% quantile and 20% quantile of the annual frequency transfer entropy matrix are taken as threshold θ to construct the network, respectively, and the constructed network is a weighted directed network. The distribution pattern of net circulation degree is consistent with that of an all-connected network. An all-connected network is less affected by extreme minimum values; thus, an analysis based on an all-connected network has certain robustness.

## 5. Discussion

In the context of globalization, the correlations among sovereign currencies are enhanced, and the differences of network characteristics of different currencies are more obvious. The network attribute reflected by this exchange rate network characteristic is undoubtedly inherent to a different sovereign currency comprehensively determined with different economic and non-economic factors in the international monetary system. As the results in Section 4.2 show, regardless of the currency, such as USD, EUR or CNY, they have different network characteristics reflecting their respective currency network attributes. The research results in Section 4.3 show that currencies with different roles in the international monetary system have different network characteristics. The dynamic characteristics of the exchange rate network reflect the evolution of the international monetary system. It is obvious that the cause-and-effect relationships among currencies can be observed by using transfer entropy to construct the exchange rate network.

The network attributes of a currency have not been concerned by the traditional exchange rate determination theory based on binary relations, while the complexity of the exchange rate determination mechanism makes it difficult to reach a consensus on the exchange rate determination theory. Although international trade and capital arbitrage are important forces driving exchange rate, the real data do not fully support the purchasing power parity theory and interest rate parity theory, and a lot of “exchange rate puzzles” have existed [36]. By considering the modern international exchange rate system as a multilateral exchange rate system, Greenaway McGreevy et al. (2018) believed that the bilateral exchange rate would be affected by some common factors, which resulted in linkage change of all exchange rates [37]. Lustig and Richmond (2020) found that the currency of a country farther away from other countries was more vulnerable to systemic currency risk on the condition that the characteristics of currency risk had been linked with physical, cultural and institutional distance [38]. It is obvious that more and more studies have paid attention to the network attribute of currency, and analysis of the characteristics of exchange rate network should be helpful for improving the exchange rate determination theory. After all, more influence should be more valuable.

It should be noted that we deal with the nominal exchange rate, while the real exchange rate is the essence of international competitiveness. The real exchange rate is approximately equal to the nominal exchange rate multiplied by the ratio of price index between the two countries. Under the condition that the ratio is stable, the real exchange rate approximately equals the nominal exchange rate multiplied by a constant. On the other hand, with floating exchange rates, the transmission of inflation from individual countries to the network may result in the formation of some average level of inflation in the network. This average level of inflation is characteristic of the entire network and does not have to blur the information carried by specific changes in real exchange rates.

At the same time, we ignore some factors worthy of consideration in further research, such as the differences of exchange rate regime. Levy-yeyati and Sturzenegger (2010) and Asici (2011) argue that there is no absolute distinction between the advantages and disadvantages of the exchange rate regime, but there should be an endogenous choice according to the economic, political and other characteristics of each country [39,40]. In reality, countries with a large number of foreign currency denominated debt and currency mismatch generally tend to choose the pegging system (hard or soft) in order to reduce losses caused by sharp exchange rate fluctuations; countries with poor reputations in the past are also more likely to choose to peg to a certain currency in order to enhance their policy credibility; and developed countries more prefer floating exchange rate regimes. The exchange rate regime will undoubtedly affect the characteristics of an exchange rate network.

## 6. Conclusions

Under the background of economic and financial globalization, the analysis of the characteristics of monetary network should not only be conducive for deepening the understanding of the evolution of international monetary system; it should also improve the exchange rate determination theory. Although it is implied that a deferent currency has similar network characteristics and plays a similar role in the global transmission of the fluctuation in the traditional exchange rate determination theory while neglecting network characteristics, every currency has different network characteristics and plays a special role, and this is observed by constructing the weighted directed network by transfer entropy based on real data. This method based on transfer entropy is effective for analyzing the causality of multidimensional exchange rate time series.

It is verified that the information flow among currencies easily surges in the period of violent market fluctuations, but the causality relationship between currencies is weakened. In the information flow network, some commodity currencies are important media in global trade. Although liquidity is poor and the volume of transactions is small, the information flow received and sent by the transit node as information transmission is large, and its role in the network cannot be ignored. These currencies with commodity attributes can be further divided into two categories: One category is the role of information source in the network, and it becomes the net sender of information in the network, such as CAD, NOK and ZAR. The other type acts as a sink in the network, such as AUD, NZD and RUB, because they are highly dependent on the outside world due to various factors. Generally speaking, commodity currency is closely related to international trade, and the role of exchange and transmission of information in the information flow network is beyond doubt. However, some commodity currencies become net issuers in the network due to the support of national strength, which can affect other currencies. Some commodity currencies have limited discourse power in the foreign exchange market, and they become net recipients and can be affected by other currencies. As information sources in the network, USD and EUR possess a greater influence on other currencies, although their reception and transmission of information flow in the network are slightly lower than that of commodity currencies. The centrality of some Asian and South American currencies is gradually improving, and the world monetary system is developing towards diversification; moreover, the currency of developing countries is becoming more and more important. In the overall time span from 2007 to 2019, CNY does not have advantages on the characteristics of influence compared with other major international currencies. However, after 2015, the dominance of CNY in the currency network gradually increased, and it was not affected by Sino–US trade friction. In terms of the comprehensive influence reflected by net flow degree, CNY surpassed Euro for the first time in 2018.

## Figures and Tables

**Table 1:** Block Classification.

Relationship Ratio within the Location	Relationship Ratio Received by Location	
	≈0	>0
≥$(g_{k}-1)/\left(g-1\right)$	Isolates	Primary
$<(g_{k}-1)/\left(g-1\right)$	Sycophants	Brokers

**Table 2:** Currencies and abbreviations of 30 countries or regions.

Currency	Abbreviation	Regions
Australian dollar	AUD	Oceania
Botswana pula	BWP	Africa
Brazilian real	BRL	South America
Canadian dollar	CAD	North America
Chilean peso	CLP	South America
Chinese yuan	CNY	Asia
Colombian peso	COP	South America
Czech koruna	CZK	Europe
Danish krone	DKK	Europe
Euro	EUR	Europe
Indian rupee	INR	Asia
Israeli new sheqel	ILS	West Asia (1)
Japanese yen	JPY	Asia
Korean won	KRW	Asia
Kuwaiti dinar	KWD	West Asia (2)
Malaysian ringgit	MYR	Asia
Mexican peso	MXN	North America
New Zealand dollar	NZD	Oceania
Norwegian krone	NOK	Europe
Polish zloty	PLN	Europe
Qatar riyal	QAR	West Asia (2)
Russian ruble	RUB	Europe
Saudi Arabian riyal	SAR	West Asia (2)
Singapore dollar	SGD	Asia
South African rand	ZAR	Africa
Swiss franc	CHF	Europe
Thai baht	THB	Asia
U.A.E. dirham	AED	West Asia (2)
U.K. pound sterling	GBP	Europe
U.S. dollar	USD	North America

**Table 3:** Weighted directed overall exchange rate threshold network node eigenvalues.

Currency	Weighted Output Degree	Weighted Input Degree	Weighted Output Closeness Centrality	Weighted Input Closeness Centrality	Betweenness Centrality	PageRank
MXN	23.97	0.00	0.028	0.000	0.000	0.033
ZAR	22.54	1.49	0.031	0.031	0.064	0.011
BWP	18.57	1.62	0.030	0.017	0.001	0.024
NOK	17.71	1.97	0.025	0.023	0.039	0.024
CAD	16.99	0.38	0.060	0.025	0.326	0.026
PLN	11.42	12.97	0.034	0.039	0.138	0.007
EUR	11.02	0.33	0.028	0.020	0.000	0.029
DKK	10.64	0.33	0.030	0.020	0.000	0.015
AUD	10.02	10.08	0.031	0.035	0.083	0.018
CZK	9.83	0.36	0.033	0.019	0.000	0.023
USD	9.39	0.53	0.037	0.017	0.000	0.052
SAR	8.85	0.73	0.034	0.018	0.000	0.023
AED	8.82	1.06	0.032	0.019	0.000	0.011
QAR	8.71	0.76	0.032	0.019	0.000	0.007
KWD	6.32	0.68	0.033	0.020	0.004	0.008
SGD	6.15	10.68	0.041	0.038	0.373	0.012
RUB	5.70	11.44	0.024	0.026	0.041	0.024
NZD	5.49	20.58	0.026	0.033	0.047	0.007
CHF	3.65	0.30	0.039	0.020	0.026	0.007
GBP	3.63	2.39	0.023	0.032	0.027	0.024
MYR	3.58	10.08	0.004	0.043	0.015	0.008
CNY	3.21	0.26	0.029	0.025	0.017	0.009
ILS	2.36	0.57	0.013	0.022	0.014	0.022
INR	2.33	3.88	0.001	0.023	0.000	0.203
JPY	1.69	1.07	0.023	0.029	0.004	0.022
THB	1.09	1.23	0.020	0.022	0.007	0.007
BRL	0.76	29.10	0.002	0.043	0.001	0.030
COP	0.37	28.78	0.003	0.042	0.014	0.007
CLP	0.31	38.92	0.031	0.032	0.128	0.229
KRW	0.00	42.53	0.000	0.038	0.000	0.035

Note: Ranking by weighted output degree value.

**Table 4:** Spillover effect analysis of currency block.

Economic Block	Relationships Accepted by the First Block	Relationships Accepted by the Second Block	Relationships Accepted by the Third Block	Relationships Accepted by the Fourth Block	Number of Members	Expected Internal Relationship Ratio	Actual Internal Relationship Ratio	Number of Relationships Received from the Outside Block	Block Classification
**the first**	23	2	1	0	9	29%	88%	139	Primary
**the second**	4	0	0	0	3	7%	0%	18	Brokers
**the third**	125	14	14	6	15	50%	9%	1	Sycophants
**the fourth**	10	2	0	0	3	7%	0%	6	Brokers

**Table 5:** The dynamic statistical characteristics of the input (output) transfer entropy of currencies.

Currency	Input Mean	Input SD	Input Skewness	Input Kurtosis	Output Mean	Output SD	Output Skewness	Output Kurtosis
AED	0.203	0.066	0.846	5.658	0.273	0.077	0.440	−0.799
AUD	0.301	0.126	1.010	3.617	0.273	0.085	0.718	−0.889
BRL	0.545	0.264	0.815	3.161	0.211	0.067	1.225	2.449
BWP	0.204	0.070	0.494	2.311	0.352	0.083	−0.525	−0.786
CAD	0.166	0.052	0.197	1.850	0.339	0.070	1.108	1.035
CHF	0.167	0.055	0.804	4.684	0.256	0.093	0.654	−0.605
CLP	0.589	0.198	0.168	2.487	0.194	0.038	0.731	−0.817
CNY	0.224	0.074	0.704	3.947	0.236	0.054	1.035	2.737
COP	0.486	0.170	0.390	3.343	0.186	0.048	0.453	0.734
CZK	0.182	0.076	0.853	2.616	0.323	0.148	0.570	−0.321
DKK	0.177	0.040	−0.843	2.362	0.329	0.148	1.430	2.499
EUR	0.163	0.044	−0.026	3.773	0.341	0.155	1.026	0.508
GBP	0.195	0.062	0.280	3.572	0.227	0.067	0.861	0.507
ILS	0.167	0.046	0.134	5.530	0.192	0.043	−0.304	−0.081
INR	0.221	0.072	0.920	5.662	0.217	0.039	−0.032	−0.839
JPY	0.221	0.087	1.155	4.732	0.241	0.089	1.223	1.790
KRW	0.614	0.199	0.276	1.433	0.174	0.055	0.870	0.642
KWD	0.186	0.048	−0.621	3.283	0.246	0.073	1.099	0.314
MXN	0.164	0.044	−0.119	3.248	0.397	0.090	0.003	0.385
MYR	0.305	0.113	0.328	2.764	0.219	0.048	0.076	−0.373
NOK	0.181	0.059	0.681	5.391	0.345	0.127	0.711	−0.639
NZD	0.449	0.263	1.875	6.409	0.242	0.054	0.358	−0.520
PLN	0.322	0.094	−0.664	2.554	0.294	0.101	0.377	−0.643
QAR	0.193	0.058	0.881	7.870	0.274	0.078	0.470	−0.852
RUB	0.330	0.185	0.906	2.893	0.236	0.042	−0.127	−1.832
SAR	0.195	0.065	1.201	8.132	0.277	0.075	0.545	−0.720
SGD	0.269	0.058	−0.939	1.927	0.268	0.069	0.679	−0.591
THB	0.206	0.043	−0.940	2.640	0.226	0.074	1.351	0.634
USD	0.196	0.069	1.253	7.528	0.284	0.072	0.688	−0.672
ZAR	0.229	0.060	−0.268	2.903	0.379	0.070	0.851	1.784

## Data Availability

https://www.imf.org/external/np/fin/ert/GUI/Pages/CountryDataBase.aspx (accessed on 13 December 2021).
